# Usage of an App-Based Addiction Prevention Program for German Vocational Students: Secondary Analysis of Data From a Cluster Randomized Controlled Trial

**DOI:** 10.2196/68754

**Published:** 2025-07-28

**Authors:** Diana Guertler, Anne Möhring, Dominic Bläsing, Christian Meyer, Hannah Schmidt, Florian Rehbein, Merten Neumann, Arne Dreißigacker, Anja Bischof, Gallus Bischof, Svenja Sürig, Lisa Hohls, Susanne Wurm, Stefan Borgwardt, Severin Haug, Hans-Jürgen Rumpf

**Affiliations:** 1 DZHK (German Centre for Cardiovascular Research), Partner Site Greifswald, Greifswald, Germany; 2 Department of Methods of Community Medicine, Institute for Community Medicine, University Medicine Greifswald, Walther-Rathenau-Str 48, Greifswald, 17475, Germany, 49 3834-867765, 49 3834-867766; 3 Helmholtz Institute for One Health, Greifswald, Germany; 4 Department of Prevention Research and Social Medicine, Institute for Community Medicine, University Medicine Greifswald, Greifswald, Germany; 5 Department of Psychiatry and Psychotherapy, University of Lübeck, Lübeck, Germany; 6 Department of Pediatrics, University of Lübeck, Lübeck, Germany; 7 Department of Social Work, FH (Fachhochschule) Münster University of Applied Sciences, Münster, Germany; 8 Criminological Research Institute of Lower Saxony (KFN), Hannover, Germany; 9 Swiss Research Institute for Public Health and Addiction, University of Zürich, Zürich, Germany

**Keywords:** eHealth, computer-tailoring, multiple addictive behaviors, vocational students, usage, mobile phone

## Abstract

**Background:**

Digital interventions have been successfully used to address addictive behaviors in adolescents and young adults. However, maintaining consistent usage remains a challenge. There is limited evidence on the determinants of usage with multiple behavioral interventions among vocational students.

**Objective:**

This paper aims to describe how vocational school students used the app-based addiction prevention program “ready4life” and to analyze student characteristics as potential determinants of intervention usage.

**Methods:**

A 2-arm cluster randomized trial evaluated “ready4life” among German vocational students aged ≥16 years. After downloading the app during class, students completed an anonymous screening and received individual risks and competencies feedback. Intervention participants (n=1286) received 4 months of individual app-based coaching, with weekly chat contacts with a conversational agent. They were asked to choose 2 of 6 modules: alcohol, tobacco, cannabis, social media and gaming, stress, and social competencies that were delivered sequentially in a random order. In addition to the weekly chats, users could self-initiate chat dialogues at any time. Chats included media (videos, images, or links), quizzes, and contests. Control participants (n=1282) received a link to health behavior information and could access coaching after 12 months.

**Results:**

Usage was low among intervention participants who received their assigned intervention (n=1266; females n=569, 44.9%; mean age 19.53, SD 3.57 years). On average, participants started 4.8 (SD 5.1) and completed 4.3 (SD 5.2) of 16 weekly in-app chats. Most students (n=903, 71.3%) completed no self-initiated chats, and 50.2% (n=636) stopped using the app before week 3. Unadjusted negative binomial multilevel regression models showed that females completed significantly more weekly dialogues (*P*<.001; incidence rate ratio [IRR] 1.55, 95% CI 1.33‐1.80), while fewer were completed by individuals with higher self-efficacy (*P*=.04; IRR 0.96, 95% CI 0.93‐0.998), higher social competencies (*P*<.001; IRR 0.97, 95% CI 0.95‐0.98), and individuals engaging in more addictive behaviors (*P*<.001; IRR 0.87, 95% CI 0.82‐0.93). Concerning specific educational tracks, professionals, technicians, associate professionals, and vocational grammar school students had the highest number of completed weekly dialogues. Determinants of completed self-initiated chats and usage time largely aligned with the findings for weekly dialogues. Additionally, those with higher perceived stress completed significantly more self-initiated chats (*P*<.001; IRR 1.19, 95% CI 1.08‐1.31). Age and year of education were not significantly associated with any of the usage parameters.

**Conclusions:**

Our study supports the existing evidence that maintaining consistent usage among adolescents and young adults is a major challenge for digital interventions. An important finding was that students with higher needs for support in terms of self-efficacy, social competence, and perceived stress showed higher intervention usage. In terms of health equity, additional efforts should be made to increase intervention usage among males, those with lower levels of education, and those with higher levels of addictive behaviors.

## Introduction

### Vocational School Students as Target Group for Addiction Prevention

Many adolescents and young adults engage in addictive behaviors, such as alcohol, tobacco, and cannabis use [1] or problematic internet use [2]. Vocational schools are promising settings for prevention efforts among adolescents and young adults [3]. First, vocational school students report even higher rates of substance use than their peers [4,5]. Second, these behaviors often tend to cluster among vocational students, making them more vulnerable to the development of noncommunicable diseases [5,6]. Representative data from German vocational students in Mecklenburg-Western Pomerania showed that 57% of vocational students had 2 or more problematic use patterns related to alcohol, tobacco smoking, cannabis, internet use, gaming, or gambling [5]. The highest clustering patterns among vocational students were found for tobacco smoking and alcohol use [5,6], and for tobacco and cannabis [5]. The clustering of addictive behavior is higher among male vocational students, those who are younger, those with lower educational attainment, and those in vocational preparation or production schools [5]. Nevertheless, vocational students differ widely in their risk profiles, that is, the combination and number of addictive behaviors. Therefore, flexible interventions are needed that can be tailored to different risk profiles.

### Usage of Digital Interventions

Digital interventions have been successfully used to prevent and reduce addictive behaviors such as substance use, among adolescents and young adults [7-9], allowing for automated delivery of highly individualized content. In particular, mobile interventions have been shown to be effective in reducing multiple addictive behaviors among vocational students [10,11].

While results regarding efficacy are encouraging, consistent intervention usage remains a challenge for digital interventions to reduce substance use in general [12,13] as well as for adolescents and young adults [8,9]. Low rates of module completion and intervention revisits are common. For example, a recent review of digital interventions to reduce substance use among adults found that on average, 60% of modules were completed, and on average, 47% of participants completed all modules [13]. In their 8-week web-based health promotion intervention for vocational students, Stassen et al [14] found that only 16.6% of all potential users logged in at least once, of which 57.4% revisited the platform. In their app-based addictive behavior intervention for vocational students, Pietsch et al [10] addressed tobacco, e-cigarettes, alcohol, and cannabis use as well as gambling and digital media-related behaviors using a voluntary commitment approach to reduce or abstain from one of these behaviors. However, only half of the students in the intervention group used the app and started a 2-week abstinence challenge.

Interventions that address multiple behaviors may add another layer of complexity. When individuals engage in multiple addictive behaviors, addressing them all at once may be overwhelming [15], potentially leading to lower usage. Previous research suggests that focusing on 2 behaviors may be optimal in terms of adherence to component recommendations [16] and intervention efficacy [15]. Additionally, completion rates are likely to differ across delivered behavior modules [17-19]. For example, in their digital lifestyle intervention, Schulz et al [17] found that completion of the smoking module was 26% compared with 47% for the alcohol module.

### Potential Determinants of Usage

Low usage is often linked to low exposure of participants to the intervention content, which in turn affects the effects seen [20,21]. Identifying predictors of usage could be used to determine specific aspects that contribute to better exposure to intervention content. A few reviews have been published on potential determinants of usage in digital interventions to reduce substance use in adult populations [12,13,22]. Intervention features that have been highlighted as important for increased usage include tailoring, reminders, customized content and features, and gamification or rewards. These were consistent with findings from a review of digital health interventions for adolescents [23]. In terms of user characteristics, the review by Jakob et al [12] found that female gender, lower substance use, and higher education were associated with higher usage in adults. Age was also associated with usage, but varied by substance: older age was associated with higher usage in digital alcohol interventions, whereas younger age was associated with higher usage in digital tobacco interventions.

To date, few studies have examined user characteristics as potential determinants of usage in digital substance use interventions among adolescents or young adults. In the mobile phone-based smoking cessation intervention for Swiss secondary and vocational school students by Paz Castro et al [24], those with a stable usage trajectory were younger, more likely to have a nonimmigrant background, and reported more perceived benefits of smoking cessation and binge drinking at baseline. Similarly, a randomized controlled trial of a mobile phone-based life-skills intervention for addiction prevention among Swiss secondary school students showed higher usage among those with lower alcohol consumption and those from upper secondary schools [25]. In addition, younger age, nonimmigrant background, and medium versus low levels of stress were associated with higher usage in that intervention in noncontrolled settings [26].

### “ready4life”: An App-Based Addiction Prevention Program for Vocational Students

The “ready4life” app, initially developed and evaluated in Switzerland [27], aims to prevent and reduce multiple addictive behaviors among vocational students and offers 6 behavior modules: alcohol, tobacco, cannabis, social media and gaming, stress, and social competencies. After receiving feedback on their individual risks and competencies in the form of a traffic light system to guide choice, users can freely choose 2 of the 6 modules to be coached in for a total of 16 weeks. Because factors such as low social competence and high work stress are likely to contribute to addictive behaviors [28,29], these topics are included as modules. We have previously shown that students have high adherence to module recommendations [30]. Usage of the “ready4life” app was stimulated by tailored content, push notifications, social and gamification features (quizzes, contests, earning credit points based on completing the weekly dialogue, or winning prizes), and personal support in the form of an “ask-the-expert” function [12]. There were weekly chats as well as self-initiated dialogues that were accessible to users at any time.

An earlier pre-post study of 5896 Swiss vocational school students provided initial insights into how students used “ready4life” [31]. At the end of the first module in week 8, only 10%‐19% still participated in the weekly chat. Self-initiated dialogues were not used at all by 61% of the students. Longer program use was observed for females and those who used more self-initiated dialogues. Other variables, such as age, self-efficacy, educational track, starting month, and delivered behavior module did not show an association with usage. “ready4life” was recently evaluated in 2 cluster randomized controlled trials among 1351 Swiss [11] and 2545 German vocational school students [32], which demonstrated its feasibility and effectiveness. Intention-to-treat comparisons showed significant positive effects of the intervention on problematic alcohol use and internet use over 6 months in the Swiss study and on social competence, stress, problematic internet use, and tobacco consumption over 12 months in the German study. However, according to the Swiss study [11], usage was rather low. On average, students in the intervention group completed 2.1 (SD 3.5) of the 16 possible weekly dialogues, with 41% completing no dialogues, and 39% completing 1 or 2 dialogues.

In summary, research on the usage of digital interventions remains scarce for both (1) vocational school students and (2) interventions targeting multiple behaviors. Specifically, only 1 study [31] has examined the determinants of intervention usage among vocational students, and there is currently no data from German samples. Given the increased vulnerability of vocational students to clustered addictive behaviors, understanding factors that enhance usage is essential.

### Aims

This study aims to add to the existing knowledge by investigating the usage of “ready4life” based on data from the German evaluation study [32,33]. Specifically, we aim to analyze student characteristics as potential determinants of usage. Based on the previous literature, usage was expected to be in the low to moderate range and to be associated with various student characteristics, such as gender.

Sociodemographics (ie, gender, age) and individual prevention needs (ie, addictive behaviors, social competence, and perceived stress) were considered as potential determinants of usage based on their relevance in previous research [12,24-26,31]. As the prevalence of addictive behaviors varies according to the educational track of vocational students [4,5], and attrition rates are likely to differ between academic and nonacademic tracks [34], we also analyzed educational track as a potential determinant of intervention usage. Self-efficacy was included because of its role in motivation and sustained effort in the behavior change process [35].

## Methods

### Design

This is a secondary analysis of data from a cluster randomized controlled trial among German vocational students testing the efficacy of the app-based addiction prevention program “ready4life.” The protocol and main results have been published elsewhere [32,33]. The trial was registered in the German Clinical Trials Register: DRKS00022328.

### Participants and Procedures

Details on the flow and characteristics of the participants can be found in Guertler et al [36]. In Germany, vocational schools are an integral part of the educational landscape, offering a variety of programs that lead to different educational and career paths. While many students participate in vocational training, which combines part-time vocational classroom instruction with practical on-the-job training, vocational schools also offer vocational preparation classes and vocational grammar school classes that allow students to earn a university entrance certificate. The sample of this study consisted of vocational students from 5 German federal states who were enrolled in vocational training, preparation, or grammar school. A total of 376 classes from 35 schools were randomized to intervention (n=186) or control (n=190) groups to avoid contamination of conditions. Between October 2020 and March 2022, classes were introduced to this study during school hours and students were invited to download the “ready4life” app. After downloading the app, all students participated in an anonymous in-app screening on their prevention needs related to alcohol, tobacco, and cannabis consumption, internet use, social competencies, and stress. Students then provided digital informed consent and contact details (email or phone number). Vocational students, aged 16+ years with smartphones who provided contact information for follow-up data collection were eligible to participate in this study. A total of 4225 app downloads were recorded, and 2568 students provided informed consent. The participation rate was 46.7% (2508/5370) among students aged ≥16 years who had the correct app version and belonged to classes where the number of students present during the introduction was known. All study participants then received in-app feedback on their individual risks and competencies in the form of a traffic light system for each behavior assessed by the in-app screening.

Students in the intervention group received 16 weeks of coaching via the “ready4life” app, as described below. The control group received only a link to information on improving health behaviors and could access the coaching after 12 months. Both study groups were invited to web-based follow-up chat sessions at 6 and 12 months via text message or email. At the end of the study, prizes were raffled, stratified by study group. In the original study [32], 1286 students from the intervention classes and 1282 from the control classes participated. In the present study, only the participants assigned to the intervention group were examined (Figure S1 in Multimedia Appendix 1).

### Intervention

**Figure 1.**
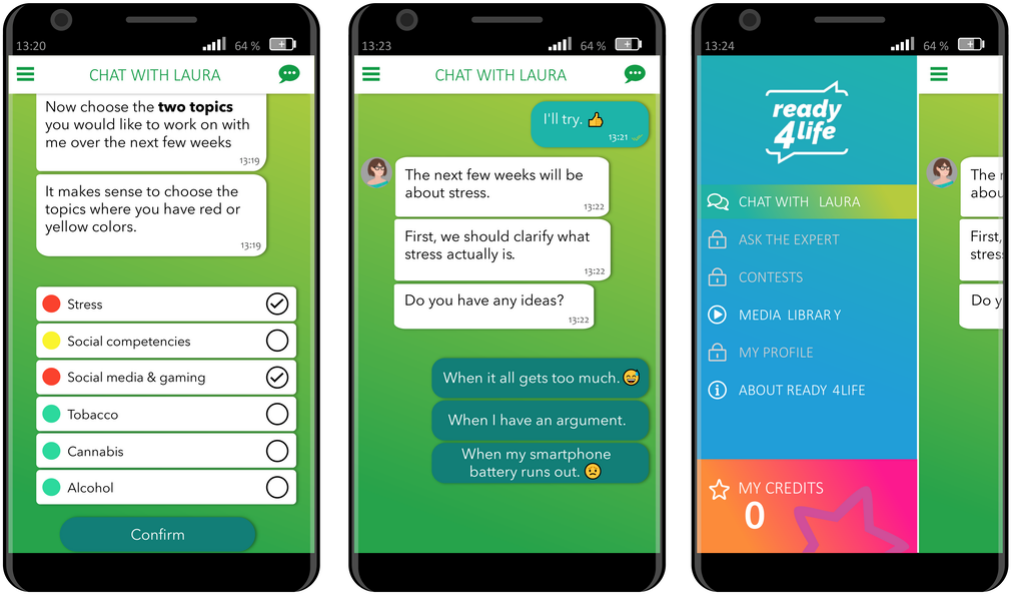
Behavior module selection, weekly chat dialogue example, and main menu (English translation).

Details on the development and content of the intervention can be found in another publication [33]. “ready4life” aims to prevent or reduce addictive behaviors and promote life skills among vocational students. Intervention participants could freely choose 2 of 6 available behavior modules: alcohol, tobacco, cannabis, social media and gaming, stress, and social competencies (Figure 1). The 2 chosen modules were presented in random order. Each module included 8 weeks of individually tailored coaching. The coaching involved weekly 5-minute chat sessions initiated by the conversational agent. The module-specific content and goals for each week can be found in the study by Schmidt et al [33]. Additionally, self-initiated chat dialogues could be started at any time after the weekly chat was completed. For each module, 4 to 5 self-initiated chats were available (Table S1 in Multimedia Appendix 1), which could be repeated as often as desired.

Chats included media (videos, images, or links), quizzes, contests, challenges, and weekly push notifications. Contests were conducted during the third week of each module. Students were encouraged to upload texts or photos showing, for example, how they have fun without a smartphone or computer (social media and gaming module) or what relaxes them when they are stressed (stress module). Uploaded content was reviewed for appropriateness by study staff. The top 3 photos with the most likes from the community were displayed. In the sixth week of each module, personal challenges were set. Students could choose 1 of several available challenges at the beginning of that week. For example, in the tobacco module, students could choose not to smoke for a while, observe smoking behavior, or help friends quit smoking. At the end of the week, the conversational agent contacted the students to ask them about their experience with the challenge. In the second week of the tobacco and alcohol module, participants could opt in to additional push notifications to receive daily tips to prepare for and support smoking cessation or low-risk alcohol consumption.

In week 5, an “ask-the-expert” feature was activated within the main menu. For 2 weeks, students could submit their questions, which were answered by a professional (eg, psychologist). The most frequently asked questions and answers were made available to everyone anonymously. Students who indicated a need for professional intervention (eg, mentioning suicidal ideation or other crises) were provided with contact information for a local, free, 24/7 telephone counseling service.

Additionally, to increase usage, participants were able to earn credit points by completing the weekly dialogue. This increased their chances of winning prizes.

### Instruments

#### Module Choice and Module Sequence

The chosen behavior modules and their random sequence were automatically recorded in the app for each student.

#### Usage

Of weekly chat dialogues, for each week, the app automatically recorded whether a participant did not start, started, or completed the chat. A chat was started if the participant had responded to the coach’s greeting at the beginning of the chat. A chat was completed if the participant had completed the chat by the time the coach said goodbye. In weeks 3 and 6, the coach contacted the participant again toward the end of the week to ask about the contest and the challenge, but no response to this second interaction was required for the chat to be counted as completed.

Of self-initiated chat dialogues, for each participant, the number and type of self-initiated chat dialogues that were started or completed were automatically recorded in the app. If a particular dialogue was started or completed multiple times, it was counted only once.

Usage time was the number of weeks between the start of the program and the last week in which a weekly chat was started or completed (possible range 0 to 16). For example, a usage time of 4 weeks would indicate that the last chat started or completed was at 4 weeks.

#### Characteristics of Vocational Students

For details on collected variables, see Guertler et al [30]. At the time of class registration, information on class level was collected, including educational track and year of education. Occupations were classified according to the International Standard Classification of Occupations 2008 [37]; Table S2 in Multimedia Appendix 1.

The app-based screening collected individual-level data on sociodemographics (age calculated from the student’s date of birth and gender), addictive behaviors, perceived stress, social competencies, and general self-efficacy.

Of the number of addictive behaviors, a total score (range: 0‐4) was created that reflects the number of addictive behaviors (problematic internet use based on the Short CIUS [38] ≥7, last month of problematic alcohol use, last month of tobacco smoking or nicotine product use, consumption of tetrahydrocannabinol-containing cannabis within the last 6 months) that a student exhibits. The last month of problematic alcohol use was defined using age- and gender-specific thresholds. For those aged ≥18 years, males had to report more than 20 drinking days or more than 2 drinks per drinking day or more than 2 maximum drinks, and females had to report more than 20 drinking days or more than 1 drink per drinking day or more than 1 maximum drink. For those aged 16 and 17, the same thresholds applied, but the threshold for drinking days was lower, at more than 10 for both males and females.

These thresholds correspond to the traffic light feedback received (yellow or red feedback; [30]).

Stress was assessed by the following question [39]: “Stress is a state in which a person feels tense, restless, nervous, or anxious, or is unable to sleep at night due to disturbing thoughts. How much do you currently feel this type of stress?” Response options ranged from 1 (“not at all strong”) to 5 (“very strong”).

Of social competencies, based on the assertion inventory [40], 8 items assessed social competence related to approaching others, expressing needs, resisting group pressure, and standing up for oneself. Responses ranged from 1 (“very uncertain”) to 5 (“very certain”), yielding a total score ranging from 8 to 40.

Of general self-efficacy, the Allgemeine Selbstwirksamkeit Kurzskala scale [41] consisted of 3 items (“I can rely on my own abilities in difficult situations,” “I am able to solve most problems on my own,” and “I can usually solve even challenging and complex tasks well”). Responses were given on a 5-point Likert scale (1 “doesn’t apply at all” to 5 “applies completely”). Based on these items, a total score ranging from 3 to 15 was calculated.

### Data Analyses

#### Software

Data were analyzed using Stata/SE (version 17.0; StataCorp LLC).

#### Usage

Of the 1286 participants in the intervention group, 20 mistyped their class password and could not access the intervention, so usage was analyzed for 1266 participants (Figure S1 in Multimedia Appendix 1). Descriptive statistics (mean and SD; median and IQR) were reported for the number of weekly and self-initiated chat dialogues started and completed, as well as for usage time. Because usage is likely to differ between the first and second module delivered, we reported descriptive usage data for the total intervention period as well as separately for the first module (week 1 to 8) and the second module (week 9 to 16). For each of the 16 intervention weeks, we reported the percentage of students who did not start, started, and completed the weekly chat dialogue.

To see if usage differed across modules, we reported usage measures for the first 8 intervention weeks stratified by the first module delivered. For each module and each self-initiated chat option, we reported the percentage of students who started and completed it at least once.

#### Potential Determinants of Usage

Multilevel negative binomial regression models were used to analyze potential determinants of (1) the number of completed weekly chat dialogues, (2) the number of self-initiated chat dialogues, and (3) usage time. Potential determinants included age, gender, educational track, year of education, number of addictive behaviors, social competence, and perceived stress. Regression models included random intercepts on the class level [42] to account for the clustered structure of the data. Intraclass correlation was calculated using an intercept-only model [43]. Intraclass correlations express the percentage of total variance in the outcome (eg, usage) that is attributable to class membership [42].

### Ethical Considerations

Ethical approval was granted by the ethics committees of the University of Lübeck (number 19‐419) and the University medicine Greifswald (BB 024/20). Digital informed consent was obtained from vocational students via the app, with no additional parental consent required under the EU General Data Protection Regulation. However, underage participants were advised to inform their parents or guardians. Students completed the app-based screening anonymously. For those who provided informed consent, research data were pseudonymized, with contact details (email or phone number) stored separately. Names were not collected; students chose nicknames. App data has been encrypted to ensure data privacy. Participants were not offered any compensation other than the chance to win prizes.

## Results

### Sample Characteristics

The sample consisted of 1266 students from the intervention group who received their assigned intervention (females n=569, 44.9%; mean age 19.53, SD 3.57 years; Table 1). Most of the students were in vocational training (n=786, 62.1%) and in their first or second year of education (n=890, 70.3%). On average, students engaged in 2.08 (SD 1.12) of 4 addictive behaviors (Table 1).

**Table 1.** Baseline description of study participants.

	Intervention group participants who received their assigned intervention (N=1266)
Gender, n (%)	
Male	697 (55.1)
Female	569 (44.9)
Age (years), mean (SD)^a^	19.53 (3.57)
Educational track, n (%)^b^^,^^c^	
Vocational training	786 (62.1)
Professionals	6 (0.5)
Technicians and associate professionals	226 (17.9)
Clerical support workers	131 (10.4)
Service and sales workers	148 (11.7)
Craft-related trades workers	205 (16.2)
Plant and machine operators and assemblers	16 (1.3)
Mixed occupations	54 (4.3)
Vocational grammar school^d^	293 (23.1)
Vocational preparation^e^	151 (11.9)
Year of education, n (%)^b^^,^^f^	
First year	515 (40.7)
Second year	375 (29.6)
Third year	119 (9.4)
General self-efficacy from 3 to 15, mean (SD)	10.85 (2.12)
Social competencies from 8 to 40, mean (SD)	29.54 (4.68)
Perceived stress from 1 to 5, mean (SD)	3.30 (1.20)
Number of addictive behaviors from 0 to 4, mean (SD)	2.08 (1.12)
Problematic internet use, n (%)^g^	902 (71.2)
Last month problematic alcohol use, n (%)^h^	841 (66.4)
Last month tobacco smoking or nicotine product use, n (%)	565 (44.6)
Consumption of tetrahydrocannabinol-containing cannabis within the last 6 months, n (%)	330 (26.1)

a Information is missing for 1 (0.1%) participant.

b Percentages do not add up to 100 due to missing information.

c Information is missing for 14 (1.1%) participants, and 22 (1.7%) students came from classes including different educational tracks.

d In Germany, most vocational schools also offer participation in vocational grammar school classes (typically grades 11 to 13) to prepare students for general university entrance certification.

e These include vocational preparation classes as well as 1- or 2-year basic training with an intermediate secondary school-leaving certificate (without training qualification).

f Information is missing for 137 (10.8%) participants, and 120 (9.5%) participants came from classes with different years of education.

g Short version of the Compulsive Internet Use Scale [38] ≥7.

h Problematic drinking in the past month was defined using age- and gender-specific thresholds. For those aged ≥18 years, males had to report more than 20 drinking days or more than 2 drinks per drinking day or more than 2 maximum drinks, and females had to report more than 20 drinking days or more than 1 drink per drinking day or more than 1 maximum drink. For those aged 16 and 17 years, the same thresholds applied, but the threshold for drinking days was lower, at more than 10 for both males and females.

### Usage

**Figure 2.**
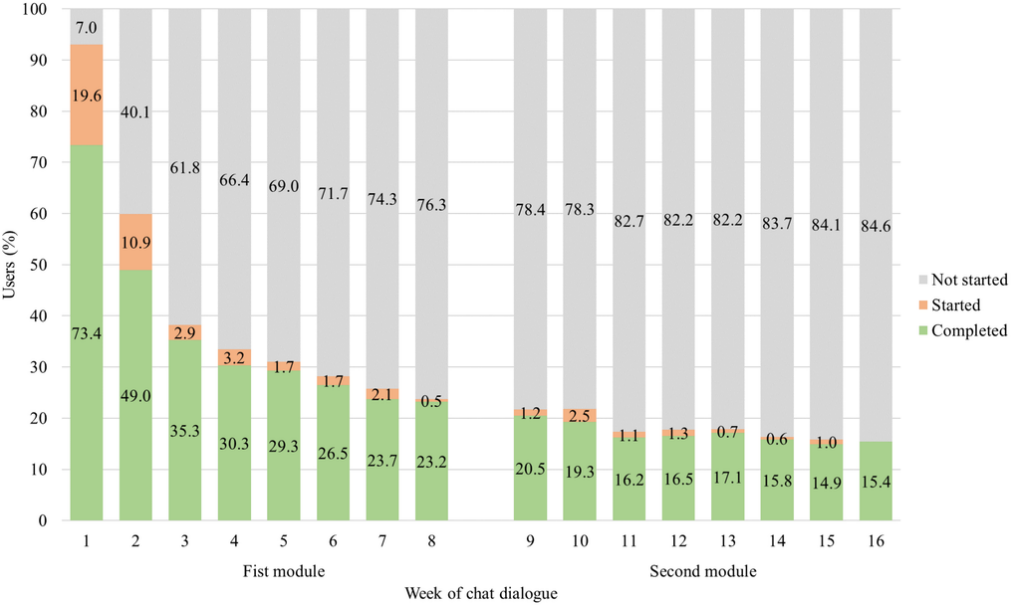
Percentage of students that had not started, started, or completed the chat dialogue per week (N=1266).

**Figure 3.**
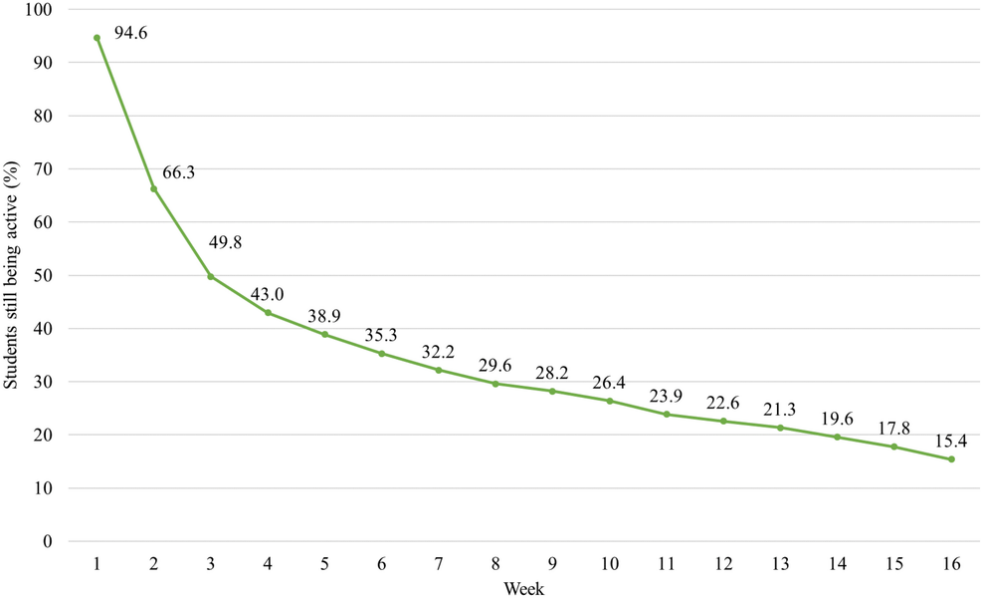
Usage over time (N=1266).

Table S3 in Multimedia Appendix 1 summarizes the usage across the sample. On average, participants started 4.8 (SD 5.1) and completed 4.3 (SD 5.2) weekly in-app chats out of the 16 possible. The median number of weekly in-app chats started was 2 (IQR 1‐7), and the median number of weekly in-app chats completed was 2 (IQR 1‐6). A total of 68/1266 (5.4%) did not start, and 264/1266 (20.9%) did not complete any of the weekly chats. However, 331/1266 (26.2%) completed one, 145/1266 (11.5%) two, 89/1266 (7%) three, 172/1266 (13.6%) four to eight, and 265/1266 (20.9%) nine or more of the weekly chats. Figure 2 shows the percentage of students who did not start, started, and completed the chat for each week separately. As can be seen, the largest drop in participation occurred between the first and second week and between the second and third week. Depending on the module combination chosen, a number of 8 to 10 self-initiated chats were available (Table S1 in Multimedia Appendix 1). Most students (903/1266, 71.3%) completed none of the self-initiated chats, 194/1266 (15.3%) completed one, and 169/1266 (13.3%) completed two or more (median 0, IQR 0‐1; mean 0.6, SD 1.2). Figure 3 illustrates usage time in terms of the percentage of students still being active over time. At the start of week 3, a total of 49.8% (630/1266) of the students were still using the app, while 50.2% (636/1266) had stopped using the app. By week 16, only 15.4% (195/1266) were still using the app. Accordingly, the median for the last weekly chat activity was 2 (IQR 1‐10) weeks, with a mean of 5.6 (SD 5.8) weeks. Usage of the first module (weeks 1 to 8) was generally higher than that of the second module (weeks 9 to 16; Table S3 in Multimedia Appendix 1).

Of the intervention group participants who received their assigned intervention, 30/1266 (2.4%) did not make a module choice. Of those who made a choice, the most commonly chosen module was stress (818/1236, 66.2%), followed by social media and gaming (625/1236, 50.6%), alcohol (360/1236, 29.1%), social competencies (306/1236, 24.8%), tobacco (232/1236, 18.8%), and cannabis (131/1236, 10.6%).

Usage during the first 8 weeks was numerically highest for the social competence module (Table S4 in Multimedia Appendix 1). Using the social competence module as a reference in multilevel negative binomial regressions, the number of started and completed weekly chats, as well as usage time, were significantly lower for the social media and gaming, alcohol, and tobacco modules, with no significant difference for the stress and cannabis modules. The results for started and completed self-initiated chats were in the same direction, but only social media and gaming and tobacco were significantly different from the social competence module (Table S5 in Multimedia Appendix 1).

### Potential Determinants of Usage

Table S6 in Multimedia Appendix 1 shows the unadjusted association of student characteristics with intervention usage. The intraclass correlations for the usage parameters ranged from 6.9% to 11.5%, indicating a modest influence of class membership on usage.

A significantly higher number of completed weekly dialogues was observed for females (*P*<.001; incidence rate ratio [IRR] 1.55, 95% CI 1.33‐1.80), while fewer were completed by individuals with higher self-efficacy (*P*=.04; IRR 0.96, 95% CI 0.93‐0.998), higher social competencies (*P*<.001; IRR 0.97, 95% CI 0.95‐0.98), and individuals engaging in more addictive behaviors (*P*<.001; IRR 0.87, 95% CI 0.82‐0.93). Concerning educational track, service and sales workers (*P*=.049; IRR 0.70, 95% CI 0.49‐0.999), and craft-related trades workers or plant and machine operators and assemblers (*P*<.001; IRR 0.57, 95% CI 0.41‐0.80) showed a lower number of completed weekly dialogues compared to professionals, technicians, and associate professionals. Clerical support workers (*P*=.16; IRR 0.76, 95% CI 0.52‐1.11), vocational grammar school students (*P*=.88; IRR 0.98, 95% CI 0.72‐1.33), and vocational preparation (*P*=.07; IRR 0.71, 95% CI 0.49‐1.03) did not differ significantly from professionals, technicians, and associate professionals in the number of completed weekly dialogues. Predictors of completed self-initiated chats and usage time largely aligned with the findings for weekly dialogues. Additionally, those with higher perceived stress completed significantly more self-initiated chats (*P*<.001; IRR 1.19, 95% CI 1.08‐1.31). Age and year of education were not significantly associated with any of the usage parameters.

The associations of social competence and the number of addictive behaviors with usage measures were independent of gender, age, and year of education (Table S7 in Multimedia Appendix 1). Self-efficacy showed a robust association with the number of self-initiated chats completed, while perceived stress showed no significant associations with any usage measure when adjusted for gender, age, and year of education. Associations between educational tracks and usage also remained, but were smaller and no longer statistically significant across all usage measures.

## Discussion

### Principal Results

This study examined how vocational school students used the app-based addiction prevention program “ready4life.” The main findings were: (1) relatively low usage, with an average of 4 of 16 (SD 5.2) weekly chats completed, and most (903/1266, 71.3%) students did not complete any self-initiated chats. About half of the students stopped using the app before the start of week three. (2) Higher usage was observed among females, individuals with lower self-efficacy, lower social competence, higher perceived stress, and fewer addictive behaviors. Additionally, professionals, technicians, and associate professionals, as well as students in vocational grammar school or vocational preparation, showed higher usage than students in training for operational and support roles.

### Interpretation of the Results and Comparison With Prior Work

#### Usage

Even though “ready4life” included several features known to increase intervention usage, such as tailored content, push notifications, and social and gamification elements [12], usage was rather low. However, low usage is consistent with the findings from a previous review of digital substance use interventions for adults [13] and substance use intervention trials among vocational students [10,14], which also indicate relatively low levels of usage.

Our findings on weekly chat and self-initiated chat use are consistent with previous “ready4life” studies. At the end of the first module in week 8, only 23.7% (300/1266) of the students in our study were still starting or completing the weekly chat, and 71.3% (903/1266) were not completing any self-initiated chats. In a previous pre-post study with 5896 Swiss vocational school students [31], only 10%‐19% started or completed the weekly chat by week 8, and 61% did not use self-initiated dialogues at all. Additionally, we found that, on average, 4.3 (SD 5.2) chats were completed, with 21% (264/1266) of students not completing any weekly chats. In a recent cluster randomized controlled trial of 1351 Swiss vocational school students [11], “ready4life” showed slightly less usage than our study, with an average of 2.1 (SD 3.5) completed weekly dialogues and 41% not completing any dialogues.

Our study found that usage varied depending on the sequence of modules, with lower usage observed for the second module. This pattern is consistent with findings from intervention studies in which multiple modules were delivered sequentially. For example, in Reinwand et al [16], participants received personalized risk feedback to guide module selection, and the modules were then delivered in a random order. They found that the more modules were recommended to be used sequentially, the lower the percentage of participants who started all of the recommended modules. When only 1 module was recommended, approximately 70% started that module. However, this percentage dropped to 30% when 2 modules were recommended.

In our study, the modules were very similar in terms of structure and type of feedback, and it may be that the dislike of repetition contributed to the lower usage of the second module. Additionally, the fact that students could not choose the order of the modules may have contributed to the low usage. For example, if a student was most interested in the stress module, but due to the random order, a less interesting module came first, this may have led to early dropout.

Another finding of our study was that usage varied depending on the behavior addressed by the module. Usage was highest for social competencies and stress modules, and lower for modules related to addictive behaviors, such as social media and gaming, alcohol, and tobacco. This is consistent with other multiple behavior interventions [17-19], showing module-specific completion rates. For example, Schulz et al [17] found lower completion rates for their smoking module (26%) compared to their alcohol (47%), physical activity (42%), vegetables (47%), or fruit (46%) modules. Brouwer et al [19] found similar results, with lower completion rates for their smoking module (58%) compared to their physical activity (89%) or fat (95%) modules. Our findings are also consistent with a recent review of mobile apps for adults, which suggested faster app abandonment for apps focusing on substance use than, for example, mental health [44]. The variability in usage based on module sequence and addressed behavior suggests that both the design and content of interventions play a critical role in participant retention and success.

#### Potential Determinants of Usage

In our study, higher usage was observed among females, individuals with lower self-efficacy, lower social competence, higher perceived stress, and fewer addictive behaviors, but also for certain educational tracks.

The higher usage among females in our study aligns with previous research showing that females are more likely to participate in health interventions [45], possibly due to higher health awareness and interest in health topics. For example, in the context of digital health information, females show higher interest in searching for such information and perceive it as more useful than males [46]. Higher self-efficacy is typically associated with greater confidence in sustaining effort and pursuing behavior change [35]. Thus, the higher usage of students with lower self-efficacy may reflect a greater need for external support or feedback. Further, the intervention may have particularly appealed to students with lower social competence (eg, more anonymity and less social pressure compared to face-to-face interventions), potentially increasing usage. Although higher usage was observed among those with higher perceived stress, this association was not independent of other factors, particularly gender. In our study, females reported higher perceived stress than males [30]. As females also showed higher usage of the intervention, this may explain the observed association between perceived stress and usage. The finding that students with more addictive behaviors used the intervention less may be explained by several factors, such as lower motivation to change, higher problem avoidance, greater feelings of being overwhelmed, or stigma. Vocational students already show low motivation to change addictive behaviors [5], which may be even more pronounced among those with multiple addictive behaviors. Engaging in multiple addictive behaviors may also reflect a higher problem severity, which has been associated with lower usage in digital interventions [25].

The fact that the highest usage was observed among professionals, technicians and associate professionals, and students in vocational grammar school may be due to the effects of socioeconomic status. In Germany, certain educational tracks are typically associated with lower socioeconomic status, for example, vocational tracks for operational and support roles (eg, service and sales workers, craft-related trades workers, plant and machine operators, and assemblers), which had the lowest usage in our study. Individuals from lower-income backgrounds often choose these vocational tracks due to financial constraints, limited access to higher education, or expectations of early entry into the labor market. These tracks typically have lower entry requirements in terms of school qualifications than professional tracks, but also offer less prestige and job security. Similarly, vocational preparation is typically attended by students with basic education, whereas vocational grammar school leads to a higher school qualification (ie, university entrance certification). Our findings highlight the problem of health inequities, whereby individuals of lower socioeconomic status have less opportunity to benefit from health behavior interventions, either because they are less likely to participate or because they used the intervention less [47], despite experiencing greater substance-related harm [48]. Lower usage among students of lower socioeconomic status may be due to lower health literacy (eg, competence to use the interventions effectively to improve health [49]), lower motivation to change, or unmet needs (eg, in terms of support or reading level). As others have argued, usage could also be influenced by differences in values, norms, knowledge, and skills [34].

Our results on determinants of usage are also consistent with a previous review [12], which found that female gender, lower substance use, and higher education were associated with higher usage of app-based substance use interventions for adults. Similarly, the aforementioned mobile phone-based life-skills intervention for addiction prevention among Swiss secondary school students showed higher usage among those with lower alcohol consumption [25] and those with moderate versus low stress levels [26]. The prepost study of “ready4life” [31] also found a longer usage time for females compared to males. In their mobile health intervention for adolescents in grades 7 to 9, Maenhout et al [34] found higher attrition during the intervention for adolescents in nonacademic educational tracks.

The fact that those with lower self-efficacy, lower social competence, and higher perceived stress used the intervention more provides evidence that the intervention is effectively reaching and potentially benefiting those who are most in need in terms of life skills, which was a major goal of the intervention. These determinants of usage align with the determinants of trial and follow-up participation in this study. For example, higher initial and follow-up participation was associated with female gender, lower social competence, higher stress, and higher education [32,36]. However, although students with lifetime cannabis consumption and higher problematic internet use were more likely to initially participate [36], those with multiple addictive behaviors may benefit less from the intervention due to lower usage. This is consistent with the fact that students with lower alcohol, tobacco, and cannabis consumption were more likely to provide complete follow-up data [32].

### Strengths

The strengths of this study include (1) targeting vocational students, an underserved group in digital intervention research, particularly in terms of usage analyses; (2) being the first study to examine determinants of usage in a multi-behavioral digital intervention for German vocational students; (3) conducting detailed analyses, including module-specific usage, multiple usage metrics, and module sequencing effects; and (4) using a facilitated access approach, which provides a more representative sample than web-based or media recruitment methods [50].

### Limitations

The results of this study should be considered in light of its limitations. First, as a secondary analysis, the original study was not designed to address the research questions posed in this paper. Second, although previous research has suggested that usage is often associated with efficacy [20,21], we have not yet tested this association in our study [51]. Third, usage was analyzed based on 3 parameters: weekly chats, self-initiated chats, and usage time. Other components, such as media content viewed, ask-the-expert, and number of logins, were not included in this analysis because the data were not automatically stored. Fourth, completion of weekly chats was rewarded with credits (and credits were tied to a prize draw), which may have influenced students’ intervention usage. Although we did not experimentally test this assumption, previous reviews have suggested this relationship (eg, [12]). Furthermore, in the focus group conducted at the end of our study, students cited the increased chances of winning prizes as a reason for continued use [52]. Fifth, findings may not be generalizable to other adolescent populations. Additionally, our previous analyses [36] showed that participants in this study differed from nonparticipants on several baseline variables, with students from nonprofessional backgrounds (eg, service and sales workers, or vocational preparation) and males being underrepresented among study participants. Conversely, those with higher support needs were overrepresented among this study’s participants, including students with lower social competence, lifetime cannabis use, higher problematic internet use, and higher perceived stress. Sixth, data collection during the COVID-19 pandemic may have influenced addictive behaviors or usage. Seventh, there may be other variables influencing usage that we have not analyzed. For example, previous studies of digital substance use interventions among adolescents or young adults found that nonimmigrant backgrounds and perceived benefits of smoking cessation [24,26] were associated with higher usage. Finally, reliance on self-report data carries the risk that the results may have been influenced by social desirability.

### Conclusions

Our study adds to the existing evidence that maintaining consistent use in adolescents and young adults is still a major challenge in digital interventions. However, despite the relatively low usage, “ready4life” was shown to be effective [32]. This is consistent with previous research suggesting that users do not necessarily need to complete the entire program to benefit [53], and that after a certain level of use, little additional benefit can be expected [54,55]. Our findings have implications for the design of multi-behavioral digital interventions. The lower usage observed for modules delivered second suggests that more relevant modules (eg, those addressing behaviors with high support needs) should be delivered first. An important finding was that students with higher needs for support in terms of self-efficacy, social competence, and perceived stress showed higher usage. In terms of health equity, additional efforts should be made to increase program usage among males, those with lower levels of education, and those with higher levels of addictive behaviors. A starting point for improving usage could be to pay more attention to the needs and wishes of these groups, for example, by involving them more closely in the development of digital interventions, or by improving usability (eg, language adaptations or supporting explanations for those with lower levels of education).

## Supplementary material

10.2196/68754 Multimedia Appendix 1 Additional figures and tables.

10.2196/68754 Checklist 1 CONSORT-EHEALTH (V 1.6.1).

10.2196/68754 Checklist 2 STROBE (Strengthening the Reporting of Observational Studies in Epidemiology) checklist.

## Abbreviations

IRR: incidence rate ratio

## References

[1] Orth B, Merkel C (2022). Der substanzkonsum jugendlicher und junger erwachsener in Deutschland, ergebnisse des alkoholsurveys 2021 zu alkohol, rauchen, cannabis und trends [Article in German]. BZgA-Forschungsbericht.

[2] Wartberg L, Kriston L, Thomasius R (2017). The prevalence and psychosocial correlates of internet gaming disorder: analysis in a nationally representative sample of 12-to 25-year-olds. Dtsch Ärzteblatt Int.

[3] Onrust SA, Otten R, Lammers J, Smit F (2016). School-based programmes to reduce and prevent substance use in different age groups: what works for whom? Systematic review and meta-regression analysis. Clin Psychol Rev.

[4] Lochbühler K, Rossa M, Ebert C, Morgenstern M, Arnaud N, Kraus L (2024). Substance use and the usage of social media, computer games, and gambling among apprentices at vocational schools. Bundesgesundheitsblatt Gesundheitsforschung Gesundheitsschutz.

[5] Meyer C, Jahnel T, Freyer-Adam J, Ulbricht S, Hanke M, John U (2016). Konsum Von Glücksspielen, Medien, Cannabis, Alkohol Und Tabak Bei Jugendlichen Und Jungen Erwachsenen in Beruflichen Schulen Und Produktionsschulen Mecklenburg-Vorpommerns: Eine Landesrepräsentative Querschnittserhebung [Gambling, Media, Cannabis, Alcohol and Tobacco Consumption Among Adolescents and Young Adults in Vocational Schools and Production Schools in Mecklenburg-Vorpommern: A State-Representative Cross-Sectional Survey] [Book in German].

[6] Atorkey P, Byaruhanga J, Paul C, Wiggers J, Bonevski B, Tzelepis F (2021). Multiple health risk factors in vocational education students: a systematic review. Int J Environ Res Public Health.

[7] Champion KE, Parmenter B, McGowan C (2019). Effectiveness of school-based eHealth interventions to prevent multiple lifestyle risk behaviours among adolescents: a systematic review and meta-analysis. Lancet Digit Health.

[8] Monarque M, Sabetti J, Ferrari M (2023). Digital interventions for substance use disorders in young people: rapid review. Subst Abuse Treat Prev Policy.

[9] Kazemi DM, Li S, Levine MJ, Auten B, Granson M (2021). Systematic review of smartphone apps as a mHealth intervention to address substance abuse in adolescents and adults. J Addict Nurs.

[10] Pietsch B, Arnaud N, Lochbühler K (2023). Effects of an app-based intervention program to reduce substance use, gambling, and digital media use in adolescents and young adults: a multicenter, cluster-randomized controlled trial in vocational schools in Germany. Int J Environ Res Public Health.

[11] Haug S, Boumparis N, Wenger A, Schaub MP, Paz Castro R (2022). Efficacy of a mobile app-based coaching program for addiction prevention among apprentices: a cluster-randomized controlled trial. Int J Environ Res Public Health.

[12] Jakob R, Harperink S, Rudolf AM (2022). Factors influencing adherence to mHealth apps for prevention or management of noncommunicable diseases: systematic review. J Med Internet Res.

[13] Shams F, Tai AMY, Kim J (2023). Adherence to e-Health interventions for substance use and the factors influencing it: systematic review, meta-analysis, and meta-regression. Digit Health.

[14] Stassen G, Grieben C, Froböse I, Schaller A (2020). Engagement with a web-based health promotion intervention among vocational school students: a secondary user and usage analysis. Int J Environ Res Public Health.

[15] Wilson K, Senay I, Durantini M (2015). When it comes to lifestyle recommendations, more is sometimes less: a meta-analysis of theoretical assumptions underlying the effectiveness of interventions promoting multiple behavior domain change. Psychol Bull.

[16] Reinwand DA, Schulz DN, Crutzen R, Kremers SP, de Vries H (2015). Who follows eHealth interventions as recommended? A study of participants’ personal characteristics from the experimental arm of a randomized controlled trial. J Med Internet Res.

[17] Schulz DN, Kremers SPJ, De Vries H (2015). Tailored eHealth lifestyle promotion: which behavioral modules do users prefer?. J Health Commun.

[18] Coumans JMJ, Oenema A, Bolman CAW, Lechner L (2021). Use and appreciation of a web-based, computer-tailored diet and physical activity intervention based on the self-determination theory: evaluation study of process and predictors. JMIR Form Res.

[19] Brouwer W, Oenema A, Raat H (2010). Characteristics of visitors and revisitors to an internet-delivered computer-tailored lifestyle intervention implemented for use by the general public. Health Educ Res.

[20] Gan DZQ, McGillivray L, Han J, Christensen H, Torok M (2021). Effect of engagement with digital interventions on mental health outcomes: a systematic review and meta-analysis. Front Digit Health.

[21] Hutton HE, Wilson LM, Apelberg BJ (2011). A systematic review of randomized controlled trials: web-based interventions for smoking cessation among adolescents, college students, and adults. Nicotine Tob Res.

[22] Milward J, Drummond C, Fincham-Campbell S, Deluca P (2018). What makes online substance-use interventions engaging? A systematic review and narrative synthesis. Digit Health.

[23] Crutzen R, de Nooijer J, Brouwer W, Oenema A, Brug J, de Vries NK (2011). Strategies to facilitate exposure to internet-delivered health behavior change interventions aimed at adolescents or young adults: a systematic review. Health Educ Behav.

[24] Paz Castro R, Haug S, Filler A, Kowatsch T, Schaub MP (2017). Engagement within a mobile phone-based smoking cessation intervention for adolescents and its association with participant characteristics and outcomes. J Med Internet Res.

[25] Paz Castro R, Haug S, Debelak R, Jakob R, Kowatsch T, Schaub MP (2022). Engagement with a mobile phone-based life skills intervention for adolescents and its association with participant characteristics and outcomes: tree-based analysis. J Med Internet Res.

[26] Haug S, Boumparis N, Wenger A, Schaub MP, Kiselev N (2023). Predictors of youth accessibility for a mobile phone-based life skills training program for addiction prevention. Int J Environ Res Public Health.

[27] Haug S, Castro RP, Wenger A, Schaub MP (2020). Efficacy of a smartphone-based coaching program for addiction prevention among apprentices: study protocol of a cluster-randomised controlled trial. BMC Public Health.

[28] Tomczyk S, Pedersen A, Hanewinkel R, Isensee B, Morgenstern M (2016). Polysubstance use patterns and trajectories in vocational students--a latent transition analysis. Addict Behav.

[29] de Jonge MC, Bukman AJ, van Leeuwen L, Onrust SA, Kleinjan M (2022). Latent classes of substance use in young adults – a systematic review. Subst Use Misuse.

[30] Guertler D, Kraft E, Bläsing D (2025). Prevention needs and target behavior preferences in an app-based addiction prevention program for German vocational school students: cluster randomized controlled trial. JMIR mHealth uHealth.

[31] Paz Castro R, Wenger A, Haug S (2021). Ready4life coaching app der lungenliga - evaluation des mobiltelefonbasierten programms zur suchtprävention bei lernenden im schuljahr 2020/21 [Ready4life coaching app of the lung league - evaluation of the mobile phone-based program for addiction prevention among students in the school year 2020/21] [Article in German].

[32] Guertler D, Schmidt H, Neumann M (2025). App-based coaching to prevent addictive behaviors in vocational school students: a cluster randomized trial. J Adolesc Health.

[33] Schmidt H, Brandt D, Bischof A (2023). App-based coaching to prevent addictive behaviors among young adults. Sucht.

[34] Maenhout L, Peuters C, Cardon G, Crombez G, DeSmet A, Compernolle S (2022). Nonusage attrition of adolescents in an mHealth promotion intervention and the role of socioeconomic status: secondary analysis of a 2-arm cluster-controlled trial. JMIR mHealth uHealth.

[35] Magill M, Meisel S, Moniz-Lewis DIK, Maisto S, Witkiewitz K (2025). Self-efficacy as a mechanism of behavior change in addiction science and practice. Curr Addict Rep.

[36] Guertler D, Bläsing D, Moehring A (2024). App-based addiction prevention at German vocational schools: implementation and reach for a cluster-randomized controlled trial. Prev Sci.

[37] Ganzeboom HB (2010). International standard classification of occupations ISCO-08 with ISEI-08 scores. Harry Ganzeboom.

[38] Besser B, Rumpf HJ, Bischof A, Meerkerk GJ, Higuchi S, Bischof G (2017). Internet-related disorders: development of the short compulsive internet use scale. Cyberpsychol Behav Soc Netw.

[39] Elo AL, Leppänen A, Jahkola A (2003). Validity of a single-item measure of stress symptoms. Scand J Work Environ Health.

[40] Gambrill ED, Richey CA (1975). An assertion inventory for use in assessment and research. Behav Ther.

[41] Beierlein C, Kovaleva A, Kemper CJ, Rammstedt B (2012). Ein messinstrument zur erfassung subjektiver kompetenzerwartungen: Allgemeine Selbstwirksamkeit Kurzskala (ASKU). Social Science Open Access Repository.

[42] Twisk JW (2006). Applied Multilevel Analysis: A Practical Guide for Medical Researchers.

[43] Leckie G, Browne WJ, Goldstein H, Merlo J, Austin PC (2020). Partitioning variation in multilevel models for count data. Psychol Methods.

[44] Kidman PG, Curtis RG, Watson A, Maher CA (2024). When and why adults abandon lifestyle behavior and mental health mobile apps: scoping review. J Med Internet Res.

[45] Oliver S, Kavanagh J, Caird J, Lorenc T, Oliver K, Harden A (2008). Health promotion, inequalities and young people’s health: a systematic review of research. EPPI Centre.

[46] Bidmon S, Terlutter R (2015). Gender differences in searching for health information on the internet and the virtual patient-physician relationship in Germany: exploratory results on how men and women differ and why. J Med Internet Res.

[47] Littlejohn C (2006). Does socio-economic status influence the acceptability of, attendance for, and outcome of, screening and brief interventions for alcohol misuse: a review. Alcohol Alcohol.

[48] Katikireddi SV, Whitley E, Lewsey J, Gray L, Leyland AH (2017). Socioeconomic status as an effect modifier of alcohol consumption and harm: analysis of linked cohort data. Lancet Public Health.

[49] Adedinsewo D, Eberly L, Sokumbi O, Rodriguez JA, Patten CA, Brewer LC (2023). Health disparities, clinical trials, and the digital divide. Mayo Clin Proc.

[50] Krause K, Guertler D, Moehring A (2019). Feasibility and acceptability of an intervention providing computer-generated tailored feedback to target alcohol consumption and depressive symptoms in proactively recruited health care patients and reactively recruited media volunteers: results of a pilot study. Eur Addict Res.

[51] Yardley L, Spring BJ, Riper H (2016). Understanding and promoting effective engagement with digital behavior change interventions. Am J Prev Med.

[52] Brandt D, Schmidt H, Gürtler D, Möhring A, Bläsing D, Meyer C (2023). Prävention bei auszubildenden in bezug auf rauschmittelkonsum und internetbezogene störungen (PARI) - abschlussbericht [prevention of drug use and internet-related disorders in trainees (PARI) - final report] [Article in German]. ready4life.

[53] Christensen H, Mackinnon A (2006). The law of attrition revisited. J Med Internet Res.

[54] Donkin L, Hickie IB, Christensen H (2013). Rethinking the dose-response relationship between usage and outcome in an online intervention for depression: randomized controlled trial. J Med Internet Res.

[55] Cunningham JA, Shorter GW, Murphy M, Kushnir V, Rehm J, Hendershot CS (2017). Randomized controlled trial of a brief versus extended internet intervention for problem drinkers. Int J Behav Med.

