# Supplementary material for: Usage of an App-Based Addiction Prevention Program for German Vocational Students: Secondary Analysis of Data From a Cluster Randomized Controlled Trial
Source: J Med Internet Res. 2025 Jul 28;27:e68754. doi: 10.2196/68754 (PMC12303402; doi:10.2196/68754)
Supplement: Multimedia Appendix 1 — Additional figures and tables. [file jmir-v27-e68754-s001.docx]

**Figure S1.** Flowchart of clusters and participants.

**Analyzed:** 1266 trial participants who received their allocated intervention from 162 classes, mean class size 7.8, range 1-28

**Analyzed:** Not applicable

Analysis

**Trial participants** (n=1286)

Excluded (n=790):

- Used wrong app version (n=146) ^b^
- Data deletion requested by user (n=7) ^c^
- Age < 16 years (n=3)
- Did not complete screening (n=273)
- Declined study participation (n=187)
- Did not deposit contact details (n=174)

**Trial participants** (n=1282)

Excluded (n=867):

- Used wrong app version (n=161) ^b^
- Data deletion requested by user (n=10) ^c^
- Age < 16 years (n=7)
- Did not complete screening (n=307)
- Declined study participation (n=210)
- Did not deposit contact details (n=172)

**Classes allocated to intervention** (n=186)

**Classes allocated to control** (n=190)

Allocation

Classes randomized (376 classes)

**Registered App Downloads** (n=2076) ^a^

**Registered App Downloads** (n=2149) ^a^

## Enrollment

**Received allocated intervention** (n=1266)

**Did not receive allocated intervention**

- Wrong password entered (n=20) ^d^

**Received allocated intervention** (n=1279)

**Did not receive allocated intervention**

- Wrong password entered (n=3) ^d^

^a^ n=11 students were identified who had registered for the study twice; these have already been subtracted from the download count.

^b^ The app included multiple versions tailored to different countries or languages. Due to a technical issue between September 2, 2021, and December 10, 2021, 307 students were mistakenly directed to a different version of the app instead of the German version. As a result, these students used a version of the app that was not part of the current study, and informed consent could not be obtained from them.

^c^ App users were able to initiate the deletion of all data collected at any time-point regardless of whether they just started the screening or provided informed consent and were using the intervention already.

^d^ Group allocation was performed on class level based on class specific passwords; due to mistyping of passwords n=20 students from the intervention group did not receive access to the intervention and n=3 students of the control groups received access to the intervention.

**Table S1.** Usage of self-initiated chat dialogues during the first eight weeks.

| Module | Chat dialogue option | Started at least once, n (%) | Completed at least once, n (%) |
| --- | --- | --- | --- |
|  |  |  |  |
| Stress (n=401) | What stress type am I? | 117 (29.2) | 114 (28.4) |
|  | What are the sources of stress and what can I do about them? | 44 (11.0) | 39 (9.7) |
|  | How does stress manifest itself in the body? | 42 (10.5) | 35 (8.7) |
|  | What are the long-term consequences of stress? | 36 (8.9) | 36 (8.9) |
|  | Stress in relationships. | 44 (11.0) | 44 (11.0) |
| Social competencies (n=151) | Taking a quiz. | 28 (18.5) | 26 (17.2) |
|  | Misunderstandings with emojis. | 26 (17.2) | 22 (14.6) |
|  | Getting to know my strengths and weaknesses. | 21 (13.9) | 10 (6.6) |
|  | Learning to be confident. | 26 (17.2) | 25 (16.6) |
|  | Speaking up about unpleasant things. | 25 (16.6) | 24 (15.9) |
| Social media and gaming (n=325) | Taking a quiz. | 96 (29.5) | 39 (12.0) |
|  | Know more about gaming and addiction. | 14 (4.3) | 7 (2.2) |
|  | Have more likes on social media! | 17 (5.2) | 16 (4.9) |
|  | Learn more about social media. | 11 (3.4) | 9 (2.8) |
|  | Learn more about cyberbullying. | 17 (5.2) | 2 (0.6) |
| Alcohol (n=188) | Taking a quiz. | 40 (21.3) | 23 (12.2) |
|  | Myth or reality? Prejudices about alcohol. | 19 (10.1) | 12 (6.4) |
|  | What effects does alcohol have on the body? | 20 (10.6) | 17 (9.0) |
|  | What is a hangover? | 24 (12.8) | 22 (11.7) |
|  | What are other people's experiences with alcohol. | 13 (6.9) | 13 (6.9) |
| Tobacco (n=108) | Taking a quiz. | 25 (23.2) | 17 (15.7) |
|  | Find out more about e-cigarettes, snus, shisha and snuff. | 9 (8.3) | 9 (8.3) |
|  | Get to know the dark side of the tobacco industry. | 10 (9.3) | 10 (9.3) |
|  | Do something for the environment. | 8 (7.4) | 8 (7.4) |
| Cannabis (n=63) | Taking a quiz. | 18 (28.6) | 13 (20.6) |
|  | Information on cannabis and addiction. | - ^a^ | 7 (11.1) |
|  | Consequences of cannabis use. | 6 (9.5) | 6 (9.5) |
|  | What is legal and what is not? | 11 (17.5) | 10 (15.9) |

^a^ Due to a technical error, the number of students who started this chat was not recorded.

**Table S2.** Classification of occupations based on ISCO-08.

| **ISCO-8 Major Groups** | **Included Occupations** |
| --- | --- |
|  |  |
| 1 Managers | Not present in the sample |
| 2 Professionals | - Applications programmers - Early childhood educators |
| 3 Technicians and associate professionals | - Administrative and executive secretaries - Chemical and physical science technicians - Civil engineering technicians - Dental assistants and therapist - Draughts persons - Information and communications technology user support technicians - Legal secretaries - Medical and dental prosthetic technicians - Nursing associate professionals - Physical and engineering science technicians - Social work associate professionals |
| 4 Clerical support workers | - Accounting and bookkeeping clerks - Bank tellers and related clerks - Clerical support workers - Contact center information clerks - General office clerks - Hotel receptionist - Secretaries - Stock clerks - Transport clerks |
| 5 Service and sales workers | - Cooks - Domestic housekeepers and housekeeping supervisors in office, hotels and other establishments - Hairdressers - Home-based personal care workers - Shop sales assistants - Waiters |
| 6 Skilled agricultural, forestry and fishery workers | Not present in the sample |
| 7 Craft-related trades workers | - Agricultural and industrial machinery mechanics and repairers - Bicycle and related repairers - Bricklayers and related workers - Building and related electricians - Cabinet-makers and related workers - Carpenters and joiners - Electrical mechanics and fitters - Electronics mechanics and servicers - Information and communications technology installers and servicers - Motor vehicle mechanics and repairers - Painters and related workers - Plumber and pipe fitters - Pre-press technicians - Precision-instrument makers and repairers - Print finishing and binding workers - Printers - Roofers - Structural metal preparers and erectors - Toolmakers and related workers |
| 8 Plant and Machine Operators and Assemblers | - Chemical products plant and machine operators - Earthmoving and related plant operators - Food and related products machine operators - Heavy truck and lorry drivers - Plastic products machine operators - Paper products machine operators - Rubber products machine operators - Plastic products machine operators - Stationary plant and machine operators |
| 9 Elementary occupations | - Cleaners and helpers in office, hotels and other establishments |
| 0 Armed forces occupations | Not present in the sample |

**Table S3.** Descriptive usage data (n=1266).

|  |  | Usage measures | | | |
| --- | --- | --- | --- | --- | --- |
|  |  | **Weekly chats** | | **Self-initiated chats ^a^** | **Usage time (in weeks)** |
|  |  | Started | Completed | Completed |  |
|  |  |  |  |  |  |
| Timeframe |  |  |  |  |  |
| Total  (week 1 to 16) | M (SD) | 4.8 (5.1) | 4.3 (5.2) | 0.6 (1.2) | 5.6 (5.8) |
|  | Median (IQR) | 2 (1-7) | 2 (1-6) | 0 (0-1) | 2 (1-10) |
| First module  (week 1 to 8) | M (SD) | 3.3 (2.7) | 2.9 (2.8) | 0.5 (1.0) | 3.7 (2.9) |
|  | Median (IQR) | 2 (1-6) | 2 (1-5) | 0 (0-1) | 2 (1-7) |
| Second module (week 9 to 16) | M (SD) | 1.4 (2.7) | 1.4 (2.6) | 0.1 (0.4) | 1.8 (3.1) |
|  | Median (IQR) | 0 (0-1) | 0 (0-1) | 0 (0-0) | 0 (0-2) |

M, Mean; SD, Standard Deviation; IQR, interquartile range.

^a^ Due to a technical error, for one self-initiated chat option in the cannabis module the number of students who started the chat was not recorded. As a result, totals were reported only for completed chats.

**Table S4.** Usage during the first eight weeks by delivered module (n=1236).

|  | Usage measures | | | | |
| --- | --- | --- | --- | --- | --- |
| Delivered module | **Weekly chats** | | **Self-initiated chats** | | **Usage time (in weeks)** |
|  | Started | Completed | Started | Completed |  |
|  |  |  |  |  |  |
| Stress (n=401) | | | | | |
| M (SD) | 3.6 (2.7) | 3.2 (2.9) | 0.7 (1.2) | 0.7 (1.2) | 4.0 (2.9) |
| Median (IQR) | 2 (1-6) | 2 (1-6) | 0 (0-1) | 0 (0-1) | 3 (1-8) |
| Social competencies (n=151) | | | | | |
| M (SD) | 4.1 (2.8) | 3.8 (3.0) | 0.8 (1.2) | 0.7 (1.1) | 4.6 (3.1) |
| Median (IQR) | 3 (2-7) | 3 (1-7) | 0 (0-1) | 0 (0-1) | 4 (2-8) |
| Social media and gaming (n=325) | | | | | |
| M (SD) | 3.2 (2.6) | 2.6 (2.8) | 0.5 (0.7) | 0.2 (0.5) | 3.6 (2.9) |
| Median (IQR) | 2 (1-5) | 1 (0-5) | 0 (0-1) | 0 (0-0) | 2 (1-7) |
| Alcohol (n=188) | | | | | |
| M (SD) | 3.0 (2.4) | 2.7 (2.5) | 0.6 (1.1) | 0.5 (1.0) | 3.4 (2.7) |
| Median (IQR) | 2 (1-4.5) | 2 (1-4) | 0 (0-1) | 0 (0-0.5) | 2 (1-6) |
| Tobacco (n=108) | | | | | |
| M (SD) | 3.0 (2.5) | 2.6 (2.5) | 0.5 (0.9) | 0.4 (0.9) | 3.4 (2.7) |
| Median (IQR) | 2 (1-4.5) | 2 (1-4) | 0 (0-1) | 0 (0-0) | 2 (1-5) |
| Cannabis (n=63) |  |  |  |  |  |
| M (SD) | 3.4 (2.9) | 3.0 (3.1) | - ^a^ | 0.6 (1.1) | 3.8 (3.0) |
| Median (IQR) | 2 (1-7) | 1 (0-6) | - ^a^ | 0 (0-1) | 2 (1-7) |

M, Mean; SD, Standard Deviation; IQR, interquartile range.

^a^ Due to a technical error, for one self-initiated chat option in the cannabis module the number of students who started the chat was not recorded.

**Table S5.** Association between usage in the first eight weeks and delivered module (n=1236).

|  | IRR (95% CI), *P* | | | | |
| --- | --- | --- | --- | --- | --- |
|  | **Weekly chats** | | **Self-initiated chats** | | **Usage time (in weeks)** |
| Delivered module | Started | Completed | Started | Completed |  |
|  | ICC = 12.3% | ICC = 10.1% | ICC = 8.7% | ICC = 7.7% | ICC = 10.8% |
|  |  |  |  |  |  |
| Stress | 0.87 (0.76-1.00),  *P* = .06 | 0.85 (0.71-10.3),  *P* = .09 | 0.87 (0.65-1.17),  *P* = .36 | 0.97 (0.68-1.37),  *P* = .85 | 0.88 (0.76-1.01), *P* = .08 |
| Social competencies | Reference | Reference | Reference | Reference | Reference |
| Social media and gaming | **0.78 (0.67-0.90), *P* < .001** | **0.69 (0.57-0.83), *P* < .001** | **0.62 (0.45-0.85), *P* = .003** | **0.34 (0.22-0.51), *P* < .001** | **0.79 (0.68-0.92), *P* = .002** |
| Alcohol | **0.75 (0.64-0.89),**  ***P* < .001** | **0.72 (0.58-0.89), *P* = .003** | 0.78 (0.55-1.11),  *P* = .17 | 0.69 (0.45-1.05),  *P* = .08 | **0.76 (0.64-0.90), *P* < .001** |
| Tobacco | **0.73 (0.60-0.89),**  ***P* < .001** | **0.69 (0.53-0.88), *P* = .003** | **0.59 (0.38-0.90), *P* = .02** | **0.59 (0.36-0.97), *P* = .04** | **0.74 (0.61-0.90), *P* = .003** |
| Cannabis | 0.85 (0.68-1.06),  *P* = .15 | 0.80 (0.59-1.07),  *P* = .13 | - ^a^ | 0.86 (0.49-1.50),  *P* = .59 | 0.85 (0.68-1.06), *P* = .15 |

Note. Displayed are results from separate multilevel negative binomial regression analyses with random intercept on class level. Significant comparisons with p < .05 are displayed as bold; IRR, Incidence rate ratio; CI, confidence interval; ICC, Intraclass-correlation; ^a^ Due to a technical error, for one self-initiated chat option in the cannabis module the number of students who started the chat was not recorded.

**Table S6.** Associations of student’s characteristics with usage parameters (n= 1266).

| Student characteristic | Weekly chats completed,  IRR (95% CI), *P* | Self-initiated chats completed,  IRR (95% CI), *P* | Usage time,  IRR (95% CI), *P* |
| --- | --- | --- | --- |
|  | ICC = 11.1% | ICC = 6.9% | ICC = 11.5% |
|  |  |  |  |
| Gender (Reference: Males) |  |  |  |
| Females | **1.55 (1.33-1.80), *P* < .001** | **1.95 (1.53-2.50), *P* < .001** | **1.43 (1.26-1.62), *P* < .001** |
| Age ^a^ | 1.02 (0.997-1.04), *P* = .09 | 1.01 (0.98-1.04), *P* = .50 | 1.01 (0.99-1.02), *P* = .38 |
| Educational track (Reference: Professionals /Technicians and associate professionals) ^b^ | | | |
| Clerical support workers | 0.76 (0.52-1.11), p = .16 | **0.48 (0.28-0.82), *P* = .007** | 0.85 (0.62-1.15), *P* = .29 |
| Service and sales workers | **0.70 (0.49-0.999), *P* = .049** | **0.40 (0.24-0.67), *P* < .001** | 0.77 (0.57-1.03), *P* = .08 |
| Craft related trades workers / Plant and machine operators, and assemblers | **0.57 (0.41-0.80), *P* < .001** | **0.58 (0.37-0.90), *P* = .02** | **0.65 (0.49-0.85), *P* = .002** |
| Vocational grammar school ^c^ | 0.98 (0.72-1.33), *P* = .88 | 0.77 (0.52-1.15), *P* = .20 | 1.05 (0.82-1.35), *P* = .71 |
| Vocational preparation ^d^ | 0.71 (0.49-1.03), *P* = .07 | 0.85 (0.52-1.37), *P* = .51 | 0.80 (0.59-1.09), *P* = .16 |
| Year of education (Reference: First year) ^e^ | | | |
| Second year | 1.09 (0.85-1.41), *P* = .50 | 1.01 (0.70-1.44), *P* = .98 | 1.07 (0.87-1.31), *P* = .51 |
| Third year | 1.07 (0.72-1.60), *P* = .73 | 0.94 (0.53-1.66), *P* = .82 | 1.11 (0.80-1.53), *P* = .53 |
| General self-efficacy | **0.96 (0.93-0.998), *P* = .04** | **0.91 (0.86-0.96), *P* < .001** | **0.97 (0.94-0.998), *P* = .03** |
| Social competence | **0.97 (0.95-0.98), *P* < .001** | **0.95 (0.93-0.98), *P* < .001** | **0.97 (0.96-0.98), *P* < .001** |
| Perceived stress | 1.05 (0.99-1.11), *P* = .12 | **1.19 (1.08-1.31), *P* < .001** | 1.05 (0.998-1.10), *P* = .06 |
| Addictive behaviors | **0.87 (0.82-0.93), *P* < .001** | **0.82 (0.74-0.91), *P* < .001** | **0.91 (0.87-0.96), *P* < .001** |

Note. Displayed are results from separate multilevel negative binomial regression analyses with random intercept on class level. ^a^ Analyses were based on n=1265 students due to missing values; ^b^ Analyses were based on n=1176 students due to missing values; ^c^ In Germany most vocational schools also offer participation in vocational grammar school classes (typically grades 11 to 13) to prepare students for general university entrance certification; ^d^ These include vocational preparation classes as well as 1- or 2-year basic training with intermediate secondary school-leaving certificate (without training qualification); ^e^ Analyses were based on n=1009 students due to missing values; Significant comparisons with p < .05 are displayed as bold; IRR, Incidence rate ratio; CI, confidence interval; ICC, Intraclass-correlation.

**Table S7.** Associations of student’s characteristics with usage parameters adjusted for gender, age and year of education (n= 1266).

| Student characteristic | Weekly chats completed,  IRR (95% CI), *P* | Self-initiated chats completed,  IRR (95% CI), *P* | Usage time,  IRR (95% CI), *P* |
| --- | --- | --- | --- |
|  | ICC = 11.1% | ICC = 6.9% | ICC = 11.5% |
|  |  |  |  |
| Educational track (Reference: Professionals /Technicians and associate professionals) ^a^ | | | |
| Clerical support workers | 0.87 (0.58-1.29), *P* = .48 | 0.59 (0.28-0.82), *P* = .05 | 0.93 (0.68-1.27), *P* = .65 |
| Service and sales workers | 0.73 (0.50-1.05), *P* = .09 | **0.44 (0.24-0.67), *P* = .002** | 0.79 (0.59-1.06), *P* = .11 |
| Craft related trades workers / Plant and machine operators, and assemblers | **0.68 (0.48-0.95), *P* = .03** | 0.77 (0.37-0.90), *P* = .26 | **0.73 (0.56-0.97), *P* = .03** |
| Vocational grammar school ^b^ | 1.09 (0.74-1.60), *P* = .65 | 0.88 (0.52-1.15), *P* = .62 | 1.18 (0.87-1.61), *P* = .28 |
| Vocational preparation ^c^ | 0.80 (0.54-1.18), *P* = .26 | 0.99 (0.52-1.37), *P* = .95 | 0.87 (0.64-1.18), *P* = .38 |
| General self-efficacy | 0.97 (0.93-1.00), *P* = .07 | **0.92 (0.86-0.98), *P* = .009** | 0.97 (0.94-1.00), *P* = .06 |
| Social competence | **0.97 (0.96-0.99), *P* = .003** | **0.96 (0.94-0.99), *P* = .01** | **0.98 (0.96-0.99), *P* < .001** |
| Perceived stress | 1.00 (0.93-1.07), *P* = .99 | 1.07 (0.96-1.20), *P* = .21 | 1.01 (0.95-1.06), *P* = .84 |
| Addictive behaviors | **0.88 (0.82-0.94), *P* < .001** | **0.84 (0.75-0.94), *P* = .002** | **0.92 (0.87-0.97), *P* = .004** |

Note. Displayed are results from separate multilevel negative binomial regression analyses with random intercept on class level adjusted for gender, age and year of education. ^a^ Analyses were based on n=1176 students due to missing values; ^b^ In Germany most vocational schools also offer participation in vocational grammar school classes (typically grades 11 to 13) to prepare students for general university entrance certification; ^c^ These include vocational preparation classes as well as 1- or 2-year basic training with intermediate secondary school-leaving certificate (without training qualification); Significant comparisons with p < .05 are displayed as bold; IRR, Incidence rate ratio; CI, confidence interval; ICC, Intraclass-correlation.
